# Efficient FIR Filter Implementations for Multichannel BCIs Using Xilinx System Generator

**DOI:** 10.1155/2018/9861350

**Published:** 2018-01-14

**Authors:** Usman Ghani, Muhammad Wasim, Umar Shahbaz Khan, Muhammad Mubasher Saleem, Ali Hassan, Nasir Rashid, Mohsin Islam Tiwana, Amir Hamza, Amir Kashif

**Affiliations:** ^1^Department of Computer Engineering, National University of Sciences and Technology, Islamabad, Pakistan; ^2^Department of Electrical Engineering, National University of Sciences and Technology, Islamabad, Pakistan; ^3^Department of Mechatronics Engineering, National University of Sciences and Technology, Islamabad, Pakistan

## Abstract

*Background*. Brain computer interface (BCI) is a combination of software and hardware communication protocols that allow brain to control external devices. Main purpose of BCI controlled external devices is to provide communication medium for disabled persons. Now these devices are considered as a new way to rehabilitate patients with impunities. There are certain potentials present in electroencephalogram (EEG) that correspond to specific event. Main issue is to detect such event related potentials online in such a low signal to noise ratio (SNR). In this paper we propose a method that will facilitate the concept of online processing by providing an efficient filtering implementation in a hardware friendly environment by switching to finite impulse response (FIR). Main focus of this research is to minimize latency and computational delay of preprocessing related to any BCI application. Four different finite impulse response (FIR) implementations along with large Laplacian filter are implemented in Xilinx System Generator. Efficiency of 25% is achieved in terms of reduced number of coefficients and multiplications which in turn reduce computational delays accordingly.

## 1. Introduction

A brain computer interface (BCI) is a communication system that allows humans to interact with their surroundings, without any involvement of nerves and muscles, by using certain control signals generated by brain that are stored in the form of electroencephalogram (EEG) [1]. BCI creates an artificial path between brain and actuated muscle that allows interaction with different devices such as computers, speech synthesizers, assistive appliances, and neural prostheses [2]. That is particularly beneficial for individuals whose muscle pathways are severely damaged by stroke. Such an interface is improving life standards for stroke patients and is also reducing intensive care [3].

**Figure 1:**
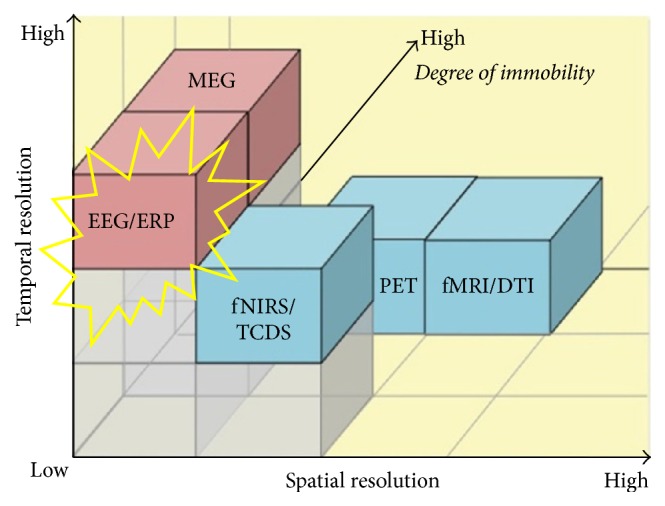
A comparison of electromagnetic (pink) and neuroimaging techniques (blue) [5].

There are different brain imaging and brain signal acquisition techniques are available that can be invasive or noninvasive. Noninvasive BCI systems do not require any type of surgery, and these are often preferred over invasive methods [4]. These techniques help in recording and visualizing brain activity which is then used to control BCIs. Some of frequently used techniques are electroencephalography (EEG), magnetoencephalogram (MEG), positron emission tomography (PET), single photon computed tomography (SPECT), functional magnetic resonance imaging (fMRI), functional near infrared spectroscopy (fNIRS), and Transcranial Direct Current Stimulation (TCDS) [5]. Every technique has its own advantages depending on the type of BCI. There are two basic criteria for selection of any techniques: (1) temporal/spatial resolution and (2) mobility index [6]. In this study we are mainly concerned with BCIs that are associated with muscle movements that require highest temporal resolution and mobility index [7]. From Figure 1 it is clear that EEG has highest temporal resolution and mobility index so we are using EEG. Other popular techniques such as fMRI and PET offer best spatial resolution [8] but they offer very bad temporal resolution [9]. fNIRS improves spatio/temporal resolution up to some extent [10] but still it cannot reach temporal resolution of EEG. So we cannot get precise muscle movement timing in our targeted BCIs for other techniques except EEG.

The EEG signals reflect a noninvasive method to record electrical activity of brain which can be termed as neurophysiology of associated task. EEG contains huge amount of data but there are certain potentials present in EEG that are specifically related to an event. Such potentials are known as event related potentials. One of the main concern of BCI system is to detect these potentials with minimum delay. But to detect these potentials from EEG is a challenging task because of very low signal to noise ratio. As EEG is prone to movement artifacts (eye blinking, etc.) and noise of different frequencies. There are different movement artifacts removal techniques that can be used to detect an activity from such noise [13]. Few popular techniques are independent component analysis (ICA), multimatching pursuit (MMP), and second-order blind identification (SOBI). For low and high frequency components present in EEG precise band pass filtering is required which is the focus of this study.

**Figure 2:**
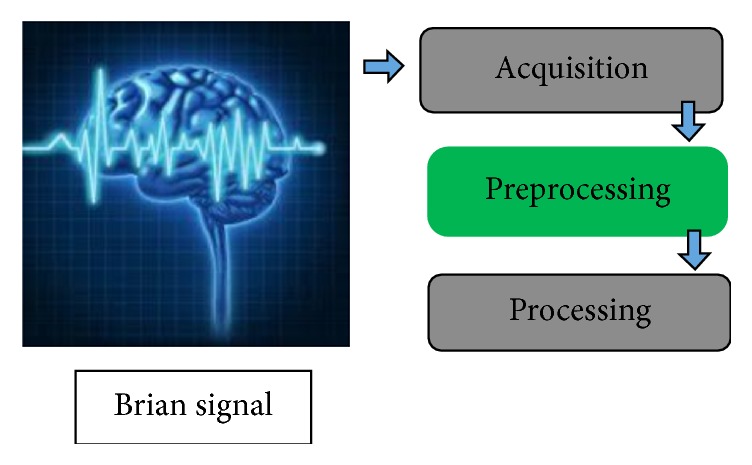
Phases of BCI (focus of study (green), not discussed (grey)).

Most BCIs consist of three major portions as shown in Figure 2. First is signal acquisition using EEG and there are certain standards for that [6, 7, 13]. We are using one hypothetical EEG signal from random dataset to validate the results. Multichannel signal which is acquired is then subjected to preprocessing to enhance SNR and remove low and high frequency components before processing [14]. We are not implementing any movement artifacts removal techniques in this study as main focus is to implement preprocessing steps (spatial filtering, band pass filtering) on Xilinx System Generator. Spatial and band pass filter are usually implemented to minimize noise and to improve SNR of the signal [15]. Depending on different applications different authors used different ranges of band pass filter along with different spatial filtering techniques [16]. All techniques are implemented in IIR domain due to sharp cutoffs of required filters. IIR filters are good tool for simulating filter response of sharp cutoff but it cannot be implemented in hardware because of feedback associated with IIR filter design [17].

We propose FIR implementation for band pass filtering along with large Laplacian spatial filtering in hardware friendly environment. Zero phase filtering is used to avoid any delays associated with FIR filter.

Four alternate methods are proposed to simulate zero phase filtering results with minimized latency in a hardware friendly environment using Xilinx System Generator:

- Flipping coefficient method
- FFT method
- Systolic Multiply Accumulator or manual filter implementation 1
- Manual filter implementation 2.

Xilinx System Generator is an efficient way for providing cosimulation environment. It is compatible with MATLAB Simulink. There is large amount of Digital Signal Processing (DSP) blocks available which includes FIR compilers, multipliers, adders, delays, and many more [12]. Black box is used for manual Verilog coding; it provides maximum flexibility but can be complex for some designs. In this study black box is used to implement large Laplacian spatial filtering. Band pass filtering is implemented using multipliers, adders, and delay blocks and dedicated FIR compiler block is used for band pass filter simulation. Performance of already given compiler and manually implemented compiler is evaluated through four different techniques.

The rest of the paper is organized in the following way. Second section is for proposed methodology. The methodology section is further divided into two subsections, first subsection explains spatial filtering and second subsection includes band pass filtering using four proposed methods. Third section explains cosimulation and selected hardware. Last section is for results and conclusion.

## 2. Methodology

### 2.1. Spatial Filtering

Multichannel data that is acquired from acquisition unit has very low SNR, so to enhance signal to noise ratio a spatial filter known as large Laplacian filter is used. This will create a single surrogate channel with enhanced SNR via localization. Different weights are assigned on the basis of channels [18].

(1) $$\begin{array}{c} x_{i}=\left\{\begin{array}{cc} 1, & i=1,\\ \frac{-1}{N_{ch}-1}, & i\neq 1. \end{array}\right. \end{array}$$

*x*_*i*_ are the weights given to different channels of EEG signal to form a single surrogate channel. *N*_ch_ depicts the number of channels. According to equation, Channel 1 was given maximum priority and maximum weight will be assigned to it.

**Figure 3:**
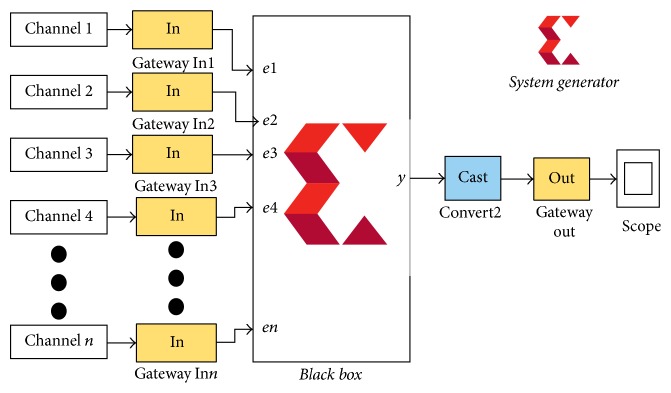
Large Laplacian in XSG to reduce SNR using localization.

In XSG this task is performed using Black Box in which a Verilog code is translated into MATLAB module as shown in Figure 3. Number of channels can vary based on the application of scalp EEG [16, 17]. *N* channel implementation is shown in Figure 3. Black box takes sample from nine channels and multiply each sample with weight and then add all these weighted samples to generate one output sample.

### 2.2. Zero Phase Band Pass Filtering

Most important phase of a BCI is band pass filtering. As EEG contains the brain activity over passage of time and contains ERPs, it contains different frequency waves (Alpha, Beta, and gamma) and abnormalities as well. So in order to detect targeted signals we limit our focus to specific frequency band via band pass filtering [17, 19].

Signals such as ERPs can be of very low potentials and lie in very narrow band. These studies require filter with very sharp cutoff due to which IIR filter is preferred but IIR cannot be implemented in hardware and FIR filter requires very large number of coefficients to achieve such cutoff. In current work focus is on very narrow band FIR filter that suffices for the requirement of narrowest range of 0.005 to 0.4 discussed in previous works [14, 16]. After computing filter coefficients zero phase filtering is done to avoid any delays associated with linear or nonlinear filtering. In Xilinx System Generator there is no block available which can perform zero phase filtering. So in this paper various methods are proposed which can perform zero phase filtering in XSG.

First MAC performs forward filtering then before using second MAC filter the output of first MAC filter is reversed and supplied to second MAC filter; this results into zero phase filtering. In XSG FIR compiler 5.0 is used for filtering; it takes FIR coefficients as parameter and filters the input signal. If we try to implement zero phase filtering using FIR compiler, then we will require two FIR compilers: first compiler will filter input signal and then before using second compiler it will be required to flip the output of first compiler. For flipping, buffering of signal will be required which will require extensive memory. An alternative to this method can be achieved using simple mathematics.

The following equations show zero phase filtering:

(2) y1=bandpass∗data,y=bandpass∗flipy1,output=flipy,output=flipbandpass∗flipy1,output=flipbandpass∗y1,

where *∗* show the convolution operation. In frequency domain the above equation can be represented as

(3) $$\begin{array}{c} OUTPUT\left(\;z\;\right)={\left(\;BANDPASS\left(\;z\;\right)\;\right)}^{2}DATA\left(\;z\;\right). \end{array}$$

Taking inverse Fourier transform we can get

(4) $$\begin{array}{c} OUTPUT=IFFT\left(\;{\left(\;BANDPASS\left(\;z\;\right)\;\right)}^{2}\;\right)data. \end{array}$$

Using mentioned equations we propose four alternative methods to achieve zero phase filtering in hardware friendly environment.

#### 2.2.1. Flipping Coefficient Method

We can achieve zero phase filtering in the following way:

 
**y** = MAC filter with original coefficients (*x*)
 
**y**2 = MAC filter with reverse coefficients (*y*).

In this method first MAC filter uses original FIR coefficients and filter sequence **x**; second MAC filter uses reverse FIR coefficients and filter sequence **y** (output of first filter).

**Figure 4:**
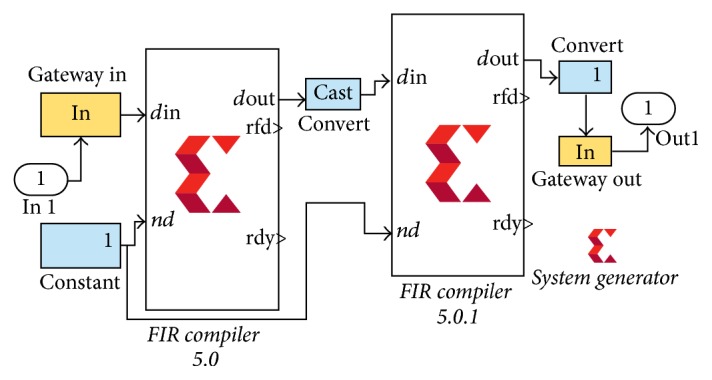
Flipping coefficients method in XSG to implement zero phase FIR filter.

Benefit of this method is reduced delay caused by flipping and buffering complete data. In Xilinx System Generator, we have to generate a dedicated block for this buffering to store complete data. But using the proposed method 1, flipping FIR coefficients offline and supplying to FIR compiler reduce delay and remove buffer. Main focus is to implement proposed methods on hardware using Xilinx System Generator (XSG). In XSG the above task is achieved by using two FIR compilers 5.0 in cascade. One compiler uses original FIR coefficients and the other FIR compiler uses Flipped FIR coefficients as shown in Figure 4.

FIR compiler provided in XSG can implement a filter up to 1024 coefficients on hardware using cosimulation file. So this technique can be verified in simulations but cannot be implemented on actual hardware.

#### 2.2.2. FFT Method

Method one uses two FIR compilers which is a waste of resources; we can perform the above task by using only one FIR compiler. If we see into the mathematics of zero phase filtering the same task can be performed by taking FFT of FIR filter coefficients, then taking square of FFT, and then performing IFFT.

**Figure 5:**
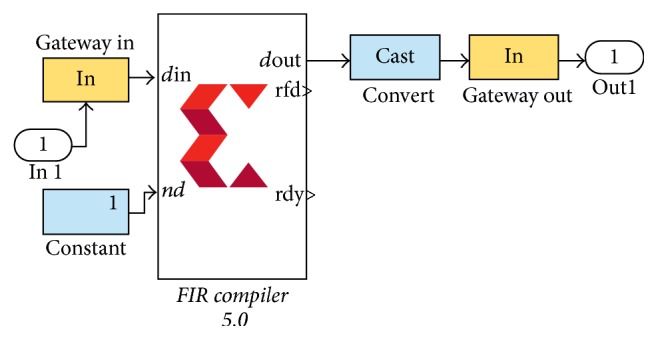
FFT method in XSG to implement zero phase band pass filtering.

Then applying MAC filter to input sequence with step 3 produced coefficients.  Figure 5 shows implementation of this method.

Same drawback as defined in previous section highlighting limitation of FIR compiler 5.0 in XSG.

#### 2.2.3. Systolic Multiply Accumulator or Manual Filter Implementation 1

**Figure 6:**
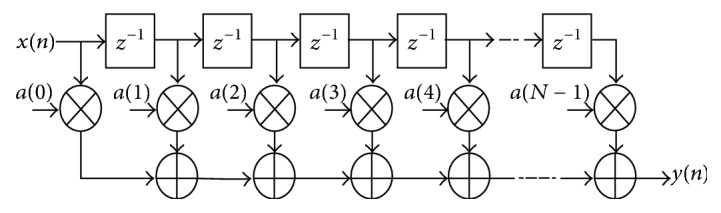
MAC filter design without symmetry [11].

FIR compiler 5.0 only works for 1024 taps. Implementing bandpass filter of 5000 taps using FIR compiler 5.0 is not possible. It works only in simulation but when we move towards hardware cosimulation it results in an error “5000 taps exceeds the limitation of FIR compiler 5.0.” Above compiler uses “Distributed Arithmetic” or “Systolic Multiply Accumulator.” Distributed Arithmetic method is very difficult to implement using basic blocks given in Xilinx System Generator library. But Systolic Multiply Accumulator can be easily implemented using basic blocks.  Figure 6 shows MAC architecture.

**Figure 7:**
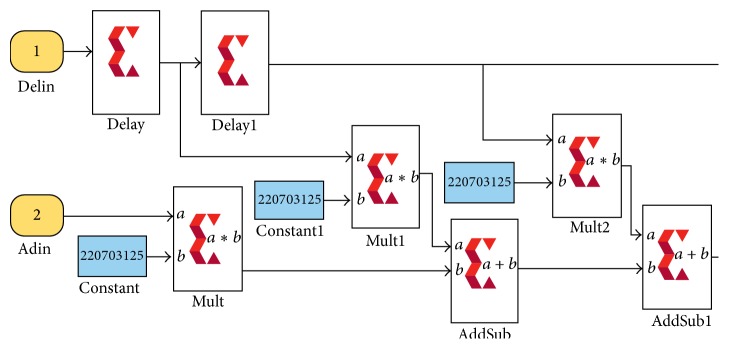
Manual filter implementation 1 in XSG using basic blocks for implementing MAC engine.

Xilinx implementation of this method is shown in Figure 7.

This manual filter implementation overcomes the limitations of predefined FIR compiler of XSG and if above architecture is implemented it will require 5000 taps to design filter of required range (0.05–0.4). In this technique 4999 adders and 5000 multipliers are used. By discarding coefficients close to zero, for this method adders are reduced to 2499 and multipliers to 2500. Utilizing symmetry of filter coefficients we move towards 2nd efficient manual implementation.

#### 2.2.4. Manual Filter Implementation 2

**Figure 8:**
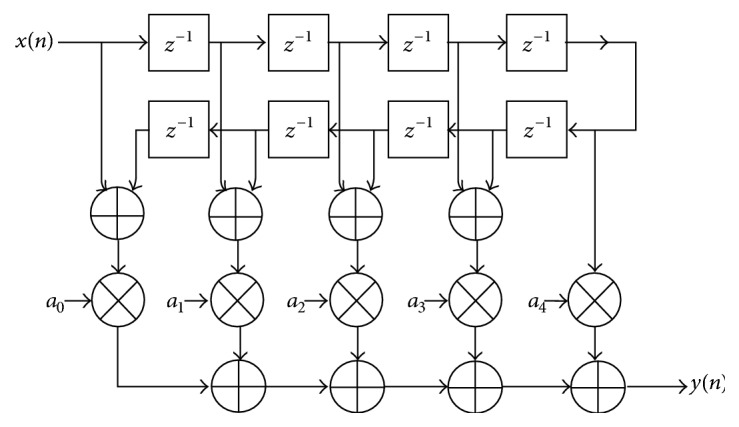
MAC filter design with symmetry [12].

**Figure 9:**
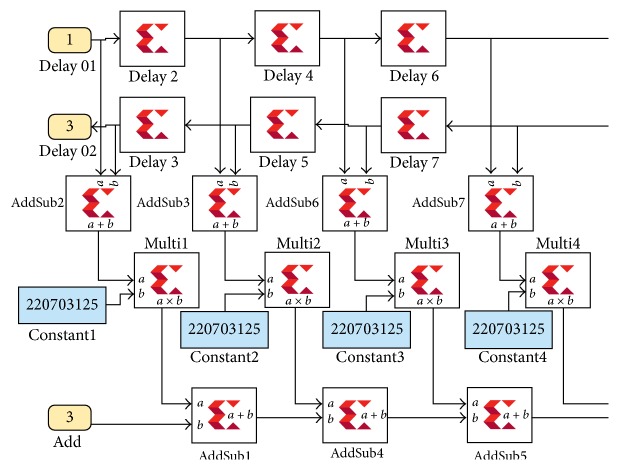
Manual filter implementation using XSG.

Instead of implementing this filter using the architecture shown in Figure 5, the more efficient signal flow-graph shown in Figure 8 can be used. In general, the former approach requires *N* multiplications and (*N* − 1) additions. In contrast, the architecture in Figure 8 requires only [*N*/2] multiplications and approximately *N* additions. This significant reduction in the computation workload can be exploited to generate efficient filter hardware. Further reduction in coefficients is achieved by discarding coefficients that are very close to zero. Filter coefficients are symmetric so we can use the following methodology. This technique uses 2499 adders and 1250 multipliers and reduced number of coefficients from 5000 to 1250.  Figure 9 shows the implementation of this method using XSG blocks.

## 3. Cosimulation Files Generation and Selected Hardware

For creating a cosimulation file, Xilinx System Generator block is used. It has many options; first option is compilation; programmer is required to select the target device for which system generator is supposed to create simulation file. In this case as there is extensive MAC engine so we have selected extreme DSP kit (vertex 4 FPGA Family). Vertex 4 FPGA has dedicated DSP slices for efficient implementation of multipliers and accumulators while Spartan family FPGAs lack this feature. In language selection option we have selected VHDL. After that by XSG it generates cosimulation files. This process takes some time depending on complexity of Simulink model. During the process it checks the model status and simulation time and then performs compilation and generation. All steps required in chip making are done, for example,  netlist generation, mapping, HDL compilation, design hierarchy analysis, and low level synthesis.

## 4. Results

**Figure 10:**
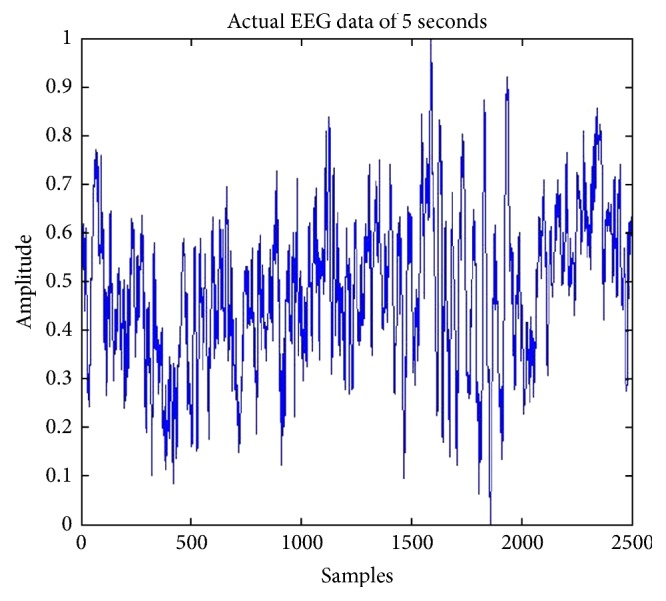
Noisy MRCP obtained from actual EEG data on MATLAB.

**Figure 11:**
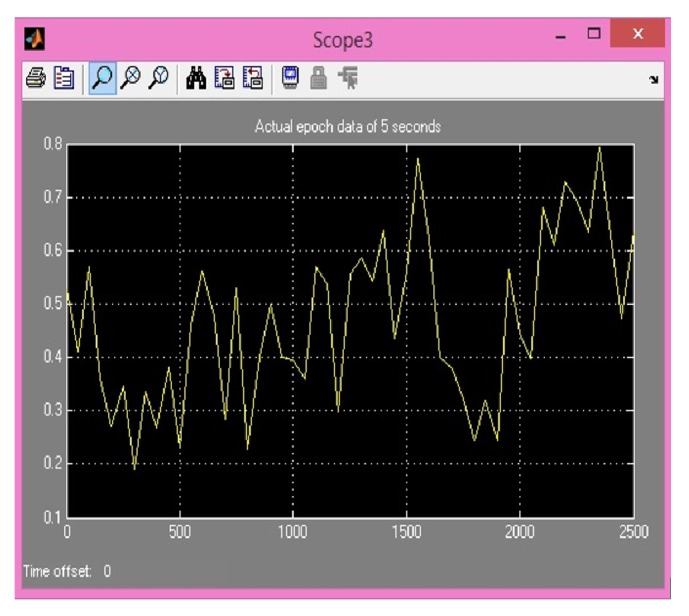
Noisy MRCP obtained from actual EEG data on XSG (samples along *x*-axis; amplitude is along *y*-axis).

**Figure 12:**
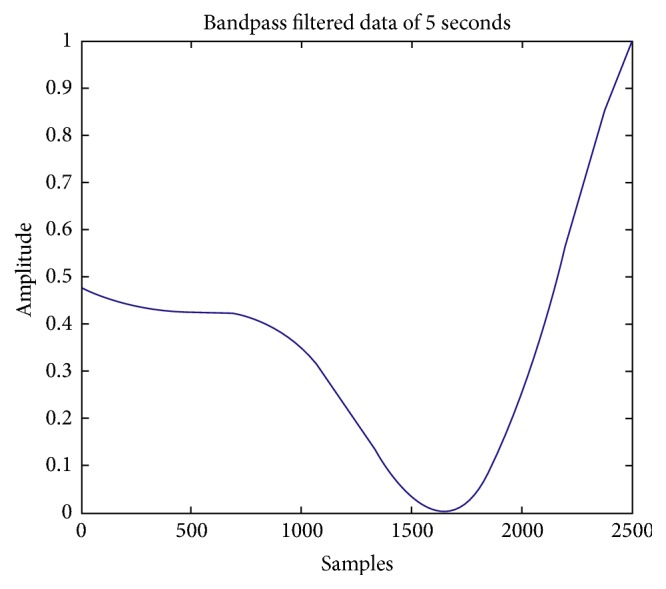
MRCP obtained from previous simulation methods.

**Figure 13:**
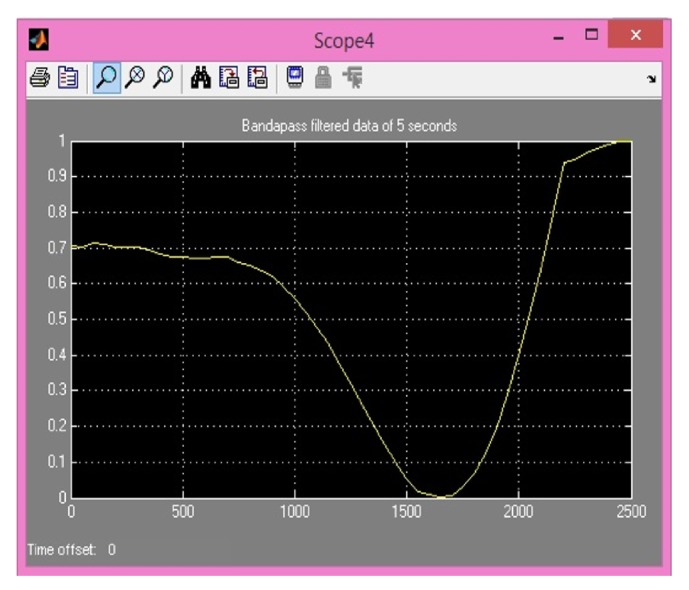
MRCP obtained from proposed method (samples along *x*-axis; amplitude is along *y*-axis).

Comparison between previously implemented techniques (using IIR) and four proposed FIR methods is shown in Table 1. Hypothetical EEG signal is used from random dataset. Then difference of preprocessing is shown by extracting an MRCP from data before and after bandpass filtering. As explained earlier MRCPs are potentials present in EEG signal between very short ranges of 0.05 Hz to 0.4 Hz [14]. Noisy extractions from EEG signal are also shown in Figure 10 for MATLAB and Figure 11 for XSG. This range is implemented in MATLAB using 2nd-order Butterworth filter. MRCPs are then extracted from EEG using bandpass filtering. Output of filtered MRCP signals using Butterworth IIR filter and proposed method is shown in Figures 12 and 13. IIR filters have definite response and are preferred for such narrow ranges. IIR filters cannot be implemented in actual hardware because of feedback coefficients. First and second proposed methods use FIR filter with 5000 coefficients to replicate the response of Butterworth IIR filter; it is implemented in simulations using two different approaches but these methods require extensive buffering and it cannot be implemented in hardware. Third method (5000 coefficients) is manual filter implementation to replicate response of first two methods but it is efficient as it does not require any buffering. Method 4 (1250 coefficients) utilizes symmetry of coefficients and analytical removal of coefficients closer to zero and its result is shown in Figure 13. Simulation results shown in Figure 13 depict that we can extract MRCPs with proposed efficient filtering technique.

Efficiency of proposed method is calculated in comparison to previous available techniques. Response of IIR filters was replicated using FIR filter and it contains 5000 coefficients which is then considered as benchmark to validate results. Our proposed method reduces the number of coefficients without any significant loss in signal and MRCP can easily be captured from this method.

(5) $$\begin{array}{c} \text{Efficiency Criteria}=100*\frac{{NC}_{p}}{{NC}_{o}}. \end{array}$$

NC_*p*_ is number of coefficients of proposed method. NC_*o*_ is number of coefficients of original method.

From Table 1  NC_*p*_ = 1250 and NC_*o*_ = 5000 so using equation we get efficiency of 25%.

Similar implication can be made for other event related potentials as well. We are using MRCP as an example but this can easily be extended for other ERPs. Proposed method with these results shows that we can move from offline filtering to real-time on device filtering.

## 5. Discussion

In this work, an effort is made to implement preprocessing steps of any BCI system that requires sharp cutoff band pass filtering in XSG to provide fast reliable hardware based filtering. Replicating response of a sharp cutoff filter in FIR domain requires very large number of coefficients. Filter used in [14, 16] is Butterworth IIR filter of range (0.05 to 0.4). FIR filter to replicate this response requires 5000 coefficients as implemented in step 1 and step 2. There are certain limitations associated with XSG [20] and it cannot process large number of coefficients required for proposed FIR filter. So an alternative method to implement sharp cutoff filters is suggested in this study (Method 3 and Method 4). If we see into the implementation perspective first two methods cannot be used to produce cosimulation results due to the filter coefficient limitation of FIR compiler 5.0. But methods 3 and 4 are implemented in hardware (VERTEX 4). We were looking to minimize the delay associated with preprocessing of BCIs and one way to reduce that delay is by reducing the calculations (multiplications and additions). In method 4 number of coefficients are reduced from 5000 to 1250 with acceptable result as shown in Figure 13. With this reduction in coefficients we claim the efficiency of 25% which will affect the computational delays as shown in Table 1 and can be considered as statistical significance of the proposed method. This also enables us to move from offline filtering towards real-time hardware based filtering with minimal delays.

## 6. Conclusion

Results show that all proposed methods produce satisfactory results in simulation environment. Method 4 is the best of all with least latency to replicate the response of IIR filters that were used in previous studies, after performing preprocessing steps in hardware environment and after validating results. In future work we will be looking into the artifacts removal techniques of EEG. Another direction is to focus on processing aspect of ERPs such as matched filtering and classification techniques.

## Figures and Tables

**Table 1:** Comparison between IIR filtering techniques and four proposed methods.

Technique	Problem	Advantage	Computational delay (300000 samples)
Butterworth IIR filters	Hardware constraints	Defined responses, better for simulations	1.22 s (MATLAB)
FIR filters	High taps	Hardware friendly, linear phase	17.4 s (MATLAB)
FIR filter (method 1)	5000 taps	Implemented in simulation environment closer to hardware	20 s (Xilinx System Generator)
FIR filter (method 2)	5000 taps	Implemented in simulation environment closer to hardware using half components as compared to method 1	13 s (Xilinx System Generator)
FIR filter (method 3)	5000 taps	Implemented on XSG via Systolic Multiply Accumulator	10 s (Xilinx System generator)
FIR filter (method 4)	1250 taps	Implemented on actual hardware via Systolic Multiply Accumulator and symmetry aspect of filter	3 s (Xilinx System generator + VERTIX 4)

## References

[1] Wolpaw J. R., McFarland D. J., Neat G. W., Forneris C. A. (1991). An EEG-based brain-computer interface for cursor control. *Electroencephalography and Clinical Neurophysiology*.

[2] Vallabhaneni A., Wang T., He B., He B. (2005). BrainComputer Interface. *Neural Engineering*.

[3] Schalk G., McFarland D. J., Hinterberger T., Birbaumer N., Wolpaw J. R. (2004). BCI2000: a general-purpose brain-computer interface (BCI) system. *IEEE Transactions on Biomedical Engineering*.

[4] Naseer N., Qureshi N. K., Noori F. M., Hong K.-S. (2016). Analysis of Different Classification Techniques for Two-Class Functional Near-Infrared Spectroscopy-Based Brain-Computer Interface. *Computational Intelligence and Neuroscience*.

[5] Mehta R. K., Parasuraman R. (2013). Neuroergonomics: A review of applications to physical and cognitive work. *Frontiers in Human Neuroscience*.

[6] Knaepen K., Mierau A., Fernandez Tellez H., Lefeber D., Meeusen R. (2015). Temporal and spatial organization of gait-related electrocortical potentials. *Neuroscience Letters*.

[7] Bulea T. C., Kim J., Damiano D. L., Stanley C. J., Park H.-S. (2015). Prefrontal, posterior parietal and sensorimotor network activity underlying speed control during walking. *Frontiers in Human Neuroscience*.

[8] Naseer N., Noori F. M., Qureshi N. K., Hong K. (2016). Determining optimal feature-combination for LDA classification of functional near-infrared spectroscopy signals in brain-computer interface application. *Frontiers in Human Neuroscience*.

[9] Baillet S., Garnero L. (1997). A Bayesian approach to introducing anatomo-functional priors in the EEG/MEG inverse problem. *IEEE Transactions on Biomedical Engineering*.

[10] Sorger B., Dahmen B., Reithler J. (2009). Another kind of ‘BOLD Response’: answering multiple-choice questions via online decoded single-trial brain signals. *Progress in Brain Research*.

[11] Chandak P. R., Giradkar V. P., Wadmalwar A. T. Design of FIR filter using Matlab Simulink and Xilinx system Generator.

[12] Athar S., Siddiqi M. A., Masud S. Teaching and research in FPGA based digital signal processing using Xilinx system generator.

[13] Snyder K. L., Kline J. E., Huang H. J., Ferris D. P. (2015). Independent component analysis of gait-related movement artifact recorded using EEG electrodes during treadmill walking. *Frontiers in Human Neuroscience*.

[14] Hassan A., Ghani U., Riaz F. (2015). Using a portable device for online single-trial MRCP detection and classification. *Lecture Notes in Computer Science (including subseries Lecture Notes in Artificial Intelligence and Lecture Notes in Bioinformatics): Preface*.

[15] Abdulkader S. N., Atia A., Mostafa M.-S. M. (2015). Brain computer interfacing: Applications and challenges. *Egyptian Informatics Journal*.

[16] Niazi I. K., Jiang N., Tiberghien O., Nielsen J. F., Dremstrup K., Farina D. (2011). Detection of movement intention from single-trial movement-related cortical potentials. *Journal of Neural Engineering*.

[17] Kamavuako E. N., Jochumsen M., Niazi I. K., Dremstrup K. (2015). Comparison of features for movement prediction from single-trial movement-related cortical potentials in healthy subjects and stroke patients. *Computational Intelligence and Neuroscience*.

[18] Karimi F., Kofman J., Mrachcz-Kersting N., Farina D., Jiang N. Comparison of EEG spatial filters for movement related cortical potential detection.

[19] Mohseni H. R., Maghsoudi A., Shamsollahi M. B. Seizure detection in EEG signals: A comparison of different approaches.

[20] LogiCORE, I. P., “FIR Compiler v5. 0, Xilinx.” San Jose, Editor,Inc., San Jose, CA, USA, 2010

